# Spontaneous Appearance and Transmission of Polydactyly in Dexter Cattle

**DOI:** 10.1155/2020/6407847

**Published:** 2020-01-16

**Authors:** Richard Browning Jr., Emily G. Hayes, Andrea S. Lear

**Affiliations:** ^1^Department of Agricultural Sciences, College of Agriculture, Tennessee State University, Nashville, TN 37209, USA; ^2^Large Animal Clinical Sciences, College of Veterinary Medicine, University of Tennessee, Knoxville, TN 37996, USA

## Abstract

A 3-yr-old Dexter cow and her yearling Dexter heifer calf exhibited polydactyly. Neither animal was linebred within 5 generations. This cow-calf pair represented the first reported occurrence of polydactyly in Dexter cattle in the US or abroad. Based upon external examination, the cow was classified as having a spontaneous unilateral case of polydactyly with an extra digit along the medial digit of the right front limb and the heifer was classified as having bilateral polydactyly because both front limbs exhibited an extra digit along the medial digit. Radiographic examination confirmed bilateral status of the heifer and revealed bilateral status of the cow. The front feet of the cow and heifer had extra bone formation consistent with an extra digit along the medial digit. Neither animal suffered from limited mobility to date or required hoof treatments. The cow produced a second calf from a different sire, a bull calf that did not appear polydactylous per external examination and was not examined radiographically. The two polydactylous animals will remain in the breeding herd to produce more study calves unless their fitness becomes compromised. Genetic aspects of the cases are discussed.

## 1. Introduction

Polydactyly is an abnormal condition in which cattle are born with one or more extra digits on one or more limbs. Polydactyly has been reported in several animal species. The condition occurs rarely in cattle but individual spontaneous cases (not produced by a known polydactylous parent) have been reported on sporadically for over a century [1, 2]. The condition has been observed in various cattle breeds [3–5]. Polydactyly is considered a congenital cause of lameness in cattle. The mode of inheritance for polydactyly has not been established in cattle, but expression of the condition varies greatly [3, 4, 6]. Euthanasia has been the course of action taken in some polydactyly cases because the severity of the foot defect(s) prevented stress-free mobility. Other cases have been of only cosmetic concern because neither animal mobility nor animal well-being were compromised. Corrective surgery has been successfully performed on some polydactylous calves [5, 7, 8]. This report presents the occurrence of an untreated, functionally normal purebred Dexter cow exhibiting polydactyly that delivered a polydactylous purebred Dexter calf. Polydactyly in Dexter cattle has not been previously reported in the scientific literature or among breeders.

## 2. Case Descriptions

**Figure 1:**
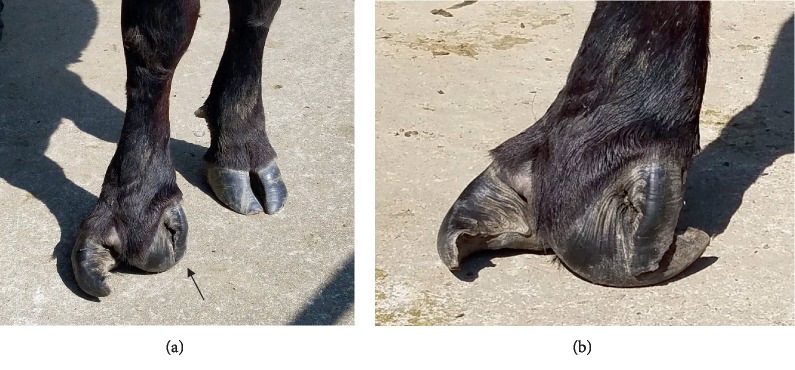
Front feet of Dexter cow Case 1 at 38 mo of age. (a) Dorsal view of feet. (b) Lateral view of right foot. The left foot appeared normal. The right foot displayed polydactyly based on a third digit alongside and detached from the abaxial wall of the medial digit. Arrow indicates the extra digit. The dew claw ipsilateral to the extra digit was absent.

A purebred Dexter heifer (Case 1) was born (23.4 kg) in a spring-born group of calves in the Tennessee State University Dexter cow herd. The heifer was the result of linecrossing with no shared ancestor between the sire and dam lines appearing through five generations. Case 1 was the product of a single-sire mating scheme. Based upon external examination, the sire and dam had normal appearing digits on all forelimbs and no other progeny from the sire (*n* = 28) or dam (*n* = 2) had obvious congenital foot abnormalities. Case 1 appeared externally normal except for her malformed right front hoof (Figure 1). The condition was first noticed when the heifer was weaned at 7 mo of age. The abaxial wall of the medial digit of the right front foot curled distal and plantar under the foot with a small, extra digit next to the medial digit and the ipsilateral dew claw missing. The lateral digit of the right front hoof grew dorsal and curled to the left at a nearly 90° angle. The right front foot digits grew divergent with a wide interdigital space. The left front foot appeared clinically normal with both dew claws present. No chronic locomotion problems were exhibited by Case 1 and she remained with her cohorts on pasture for first breeding.

**Figure 2:**
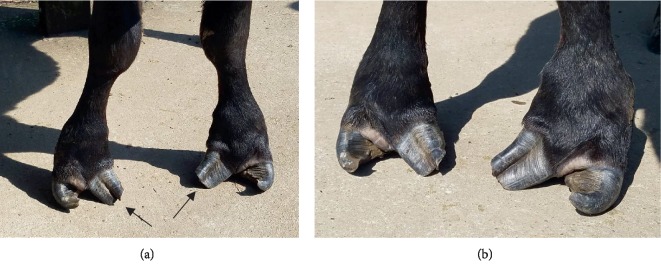
Dorsal view of the front feet of Dexter heifer Case 2 at 14 mo of age. (a) Lower limb view showing distorted leg structure. (b) Close-up of feet showing bilateral polydactyly based a third digit fused to the abaxial wall of each medial digit. Arrows indicate the notches or ridges on the hoof wall demarcating fused digits. All dew claws on these feet were present.

At 24 mo of age, Case 1 delivered her first calf, a purebred heifer (Case 2) born (23.4 kg) without assistance. The sire of Case 2 was a Dexter bull that was linebred for the same common bull ancestor as the maternal granddam of Case 2 (dam of Case 1), but from a different family line. There were no shared ancestors between the sire and dam lines appearing through five generations of Case 2. Case 2 was externally normal except for both front feet being malformed with an extra, fused digit visible on the abaxial wall of each medial digit and pronounced interdigital space (Figure 2). The malformed feet were first noticed at 24 h of age. All dew claws were present for Case 2. Despite the abnormality, Case 2 was able to move with the herd and suckled her dam on pasture until self-weaning by 10 mo of age.

At 34 mo of age, Case 1 delivered her second calf, a purebred bull (24.9 kg) sired by a Dexter bull from the same linebred family as the dam of Case 1. The 5-generation inbreeding coefficient was 0.15 for the bull calf. He was clinically normal with no hoof abnormalities based on external examination at one mo of age.

**Figure 3:**
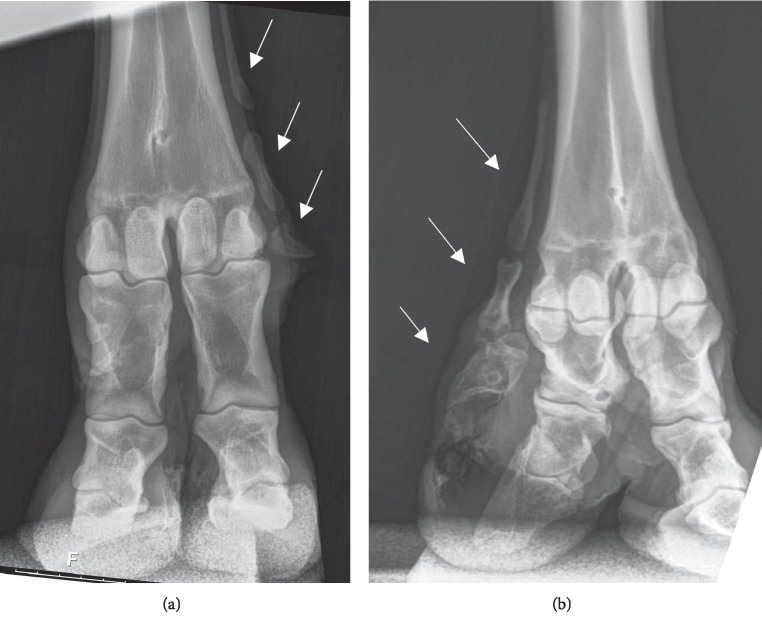
Palmodorsal radiographs of the front feet of Dexter cow Case 1 at 35 mo of age. (a) Left front foot. (b) Right front foot. Both feet presented segmented bone development (shown by the arrows) indicating an extra digit next to the medial digits. Arrows indicate extra bone development. The extra digit structure on the right foot extended to the area of the third phalanx. The extra digit structure on the left foot extended to the area of the sesamoid bones and dew claw.

A radiographic examination of the forelimbs for Case 1 was performed at 35 mo of age. The animal was held in standing restraint in a cattle chute. Lateral and palmo-dorsal images were taken of both distal forelimbs using a digital radiographic unit (Next Equine DR Unit; Sound, Carlsbad CA USA). Radiographic examination of the front right foot (Figure 3) revealed segmented bone development extending from the metacarpal area of the limb to the distal phalanx and likely represented digit II and associated with the extra digit evident along the abaxial wall of the medial digit. Radiograph of the front left foot exposed segmented bone development along the medial toe that ended at the level of the sesamoid bones and in the proximity of the medial dew claw. It was thought that the extra bone structure was representative of digit II development. Externally, Case 1 was viewed as being a unilateral case, but the front left foot radiograph led to the cow being reclassified as a bilateral case of polydactyly. Subsequently, the extra bone growth on the front left limb was found to be detectable by manual palpation. Manual palpation did not indicate the presence of extra bone growth on either limb of her bull calf and no radiographs were taken of him.

**Figure 4:**
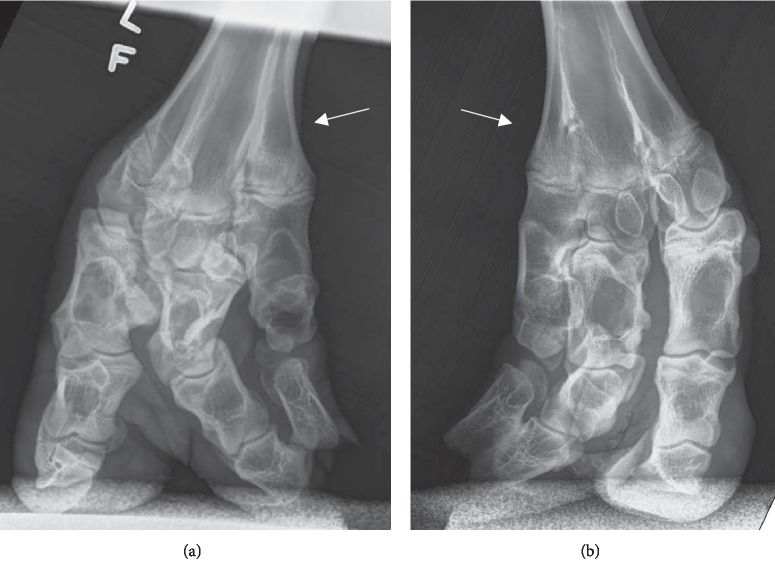
Palmodorsal radiographs of the front feet of Dexter heifer Case 2 at 11 mo of age. (a) Left front foot. (b) Right front foot. Both feet presented segmented bone development indicating an extra digit next to each medial digit. Each foot presented an extra fused section of the metacarpus (shown by the arrows). The extra digits extended the length of the foot with apparent fusion with the third phalanx of the normal digits.

Radiographs were performed on Case 2 at 11 mo of age as described above and showed similar development for the front medial digits, bilaterally (Figure 4). An extra fused section of the metacarpus was visible on each foreleg with disjointed phalangeal bones present, likely representing digit II formation and associated with the extra toe section evident on the abaxial hoof wall of the medial digits. The distal phalanx of digit III seemed to be fused with the distal portion of the digit II structure for both medial digits.

## 3. Discussion

Polydactyly in this cow-calf pair seems to be the first reported occurrence in Dexter cattle. No other cases of polydactyly have been produced in the Tennessee State University herd of Dexter cattle among 161 calves over 4 years of breeding. Dexter has not been identified in past published reports of polydactyly among cattle breeds [3–6]. Inquiries to noted breed historians did not reveal any communicated cases within Dexter herds in the US, England, or Australia. The Dexter breed is noted for chondrodysplasia, a skeletal malformation presented in the form of lethal bulldog dwarfism [9]. Mutated aggrecan alleles [10] for this lethal genetic condition persist in the US Dexter cattle population because some breeders seek to produce chondrodysplasia allele carriers to preserve the heterozygous dwarfism phenotype historically significant to the breed. The University herd does not maintain chondrodysplasia carriers, so mutated aggrecan alleles were not associated with the polydactyly observations reported here.

Few bovine case reports have presented parent-progeny occurrences of polydactyly. Roberts [2] demonstrated three successive generations of polydactyly in Holstein cattle including matings of a polydactylous Holstein cow to an unrelated normal Holstein bull that produced three polydactous bull calves, but no polydactylous heifer calves. Johnson et al. [6] conducted a series of breeding trials involving mostly polydactylous cattle mated with a parent to test modes of polydactyly inheritance, but included two polydactylous Simmental cows crossbred with a normal Hereford bull that produced one polydactylous male fetus, no polydactylous female fetuses and six normal fetuses. The current case adds to past pedigree assessments [2, 6] by showing that a polydactylous cow (Case 1) can produce a polydactylous heifer calf (Case 2) without using a parent-offspring inbred mating. Roberts [2] had a normal bull x polydactylous cow mating produce a polydactylous heifer calf, but the genetic relationship between the sire and dam was not disclosed. The occurrence of a polydactylous bull siring a polydactylous heifer calf has also been reported [7]. The current cow-calf pair of Case 1 and Case 2 supported the general position that polydactyly can be genetically transmitted from parent to offspring in cattle.

Various modes of inheritance have been considered for bovine polydactyly, but none have been validated [6, 11, 12]. The pedigree pattern of Roberts [2] suggested a clear case of simple dominance, whereas Johnson et al. [6] ruled out simple dominance along with other possible modes. The occurrence of polydactyly in Case 1 as a product of linecrossing aligned with polydactylous Holstein calves having low inbreeding coefficients [13, 14]. Thus spontaneous bovine cases have not always been a product of intensive inbreeding. It is not clear if polydactyly in Case 1 and other spontaneous cases have resulted from the coupling of alleles latent in breed populations and persisting at very low frequencies or the result of spontaneous, possibly recurring, mutations associated with zygote formation and conceptus development. A polygenic mode of inheritance as previously suggested [6] supports the possibility of latent alleles. However, simple dominance of a mutated allele cannot be ruled out.

Preliminary work [14] identified two new gene mutations in a sampled Holstein population associated with polydactyly; the mutations were not found in other breeds screened. Generally, recurrent *de novo* mutations as opposed to latent alleles may help to explain spontaneous polydactyly cases reported sporadically over time and the past difficulties of defining the mode of inheritance [15]. The possibility of recurring *de novo* mutations of either prezygotic germline or postzygotic type and the lack of a genetic marker test for polydactyly makes it difficult to make sound culling decisions on parents of a spontaneous polydactylous calf because the parents may both be normal noncarriers. Applying the family planning discussion of Acuna-Hidalgo et al. [15] for recurring *de novo* mutations, the risk of a second spontaneous polydactylous calf from the same sire-dam mating is very low, but somewhat dependent on parental age with emphasis on older sires.

The clinical presentation of the left front limb of Case 1 offered an interesting scenario because of the hidden phenotypic expression of the extra digit formation. As such, Case 1 was originally classified as unilateral until the radiograph revealed otherwise. It is possible that some instances of polydactyly may go undetected because the hooves appear normal externally. The presentation of polydactyly was much more pronounced at the skeletal level for Case 2 than for her dam. Positioning of the distal end of extra digit bone structure relative to dew claw presence or absence suggested that the dew claw was associated with the extra digits in Case 1 and not associated with the extra digits in Case 2. The varied expression of polydactyly in cattle is probably influenced by random embryonic tissue responses to gene mutations during limb differentiation [4]. Cases of “hidden” polydactyly could confuse efforts to define the mode of inheritance.

The cow and heifer calf subjects of this report will remain in the University breeding herd as long as mobility is not impaired and well-being is not compromised. To date neither animal has required hoof treatments, although this may change as they grow to maturity. In consultation with and upon recommendation of one of the herd veterinarians, a case animal will be euthanized when lameness and compromised well-being become evident. Possible future matings of Case 1 and Case 2 may provide more information about transmission and expression of bovine polydactyly. In a commercial setting, polydactylous cattle not chronically lame can remain in the herd as terminal market animals [16]. Using a polydactylous bull or cow as breeding stock, including those receiving corrective surgery, should be avoided because progeny may inherit polydactyly and the level of expression in calves, from superficial to debilitating, cannot be predicted. The economic loss caused by euthanasia of a debilitated calf or the blemished reputation of a genetic line of seedstock producing a polydactylous calf should not be overlooked.
